# Garcinoic acid prevents β-amyloid (Aβ) deposition in the mouse brain

**DOI:** 10.1074/jbc.RA120.013303

**Published:** 2020-07-02

**Authors:** Rita Marinelli, Pierangelo Torquato, Desirée Bartolini, Cristina Mas-Bargues, Guido Bellezza, Antimo Gioiello, Consuelo Borras, Antonella De Luca, Francesca Fallarino, Bartolomeo Sebastiani, Sridhar Mani, Angelo Sidoni, Jose Viña, Manuela Leri, Monica Bucciantini, Pamela Nardiello, Fiorella Casamenti, Francesco Galli

**Affiliations:** 1 Department of Pharmaceutical Sciences, University of Perugia, Perugia, Italy; 2 Freshage Research Group, Dept. of Physiology, Faculty of Medicine, University of Valencia, CIBERFES, INCLIVA, Valencia, Spain; 3 Department of Experimental Medicine, Section of Anatomic Pathology and Histology, Medical School, University of Perugia, Perugia, Italy; 4 Department of Experimental Medicine, University of Perugia, Perugia, Italy; 5 Departments of Medicine, Genetics and Molecular Pharmacology, Albert Einstein College of Medicine, Bronx, New York USA; 6 Department of Experimental and Clinical Biomedical Sciences, University of Florence, Italy; 7 Department of Neuroscience, Psychology, Drug Research and Child Health, University of Florence, Italy

**Keywords:** neurodegenerative disease, garcinoic acid, vitamin E, peroxisome proliferator-activated receptor gamma (PPARγ), pregnane X receptor (PXR), apolipoprotein E (ApoE, ), genistein, Alzheimer's disease, protein aggregation, tocopherol, tocotrienol, peroxisome proliferator-activated receptor (PPAR), Alzheimer disease, amyloid-beta (AB)

## Abstract

Garcinoic acid (GA or δ-T3-13'COOH), is a natural vitamin E metabolite that has preliminarily been identified as a modulator of nuclear receptors involved in β-amyloid (Aβ) metabolism and progression of Alzheimer's disease (AD). In this study, we investigated GA's effects on Aβ oligomer formation and deposition. Specifically, we compared them with those of other vitamin E analogs and the soy isoflavone genistein, a natural agonist of peroxisome proliferator–activated receptor γ (PPARγ) that has therapeutic potential for managing AD. GA significantly reduced Aβ aggregation and accumulation in mouse cortical astrocytes. Similarly to genistein, GA up-regulated PPARγ expression and apolipoprotein E (ApoE) efflux in these cells with an efficacy that was comparable with that of its metabolic precursor δ-tocotrienol and higher than those of α-tocopherol metabolites. Unlike for genistein and the other vitamin E compounds, the GA-induced restoration of ApoE efflux was not affected by pharmacological inhibition of PPARγ activity, and specific activation of pregnane X receptor (PXR) was observed together with ApoE and multidrug resistance protein 1 (MDR1) membrane transporter up-regulation in both the mouse astrocytes and brain tissue. These effects of GA were associated with reduced Aβ deposition in the brain of TgCRND8 mice, a transgenic AD model. In conclusion, GA holds potential for preventing Aβ oligomerization and deposition in the brain. The mechanistic aspects of GA's properties appear to be distinct from those of other vitamin E metabolites and of genistein.

Alzheimer's disease (AD) is a progressive neurodegenerative disorder characterized by the deposition of Aβ (β-amyloid) plaques and tau neurofibrillary tangles, and by the development of neuroinflammation and increased neuronal cell death (1).

PPARγ is a ligand-activated transcription factor implicated in glucose and lipid metabolism with a known regulatory role in AD pathogenesis, thus it has been recently investigated in a therapeutic approach (2). Among its activity PPARγ stimulate the expression of a cholesterol transport protein apolipoprotein E (ApoE), which is involved in the clearance of the soluble forms of β-amyloid peptides (Aβ). Such an important function has been proposed to prevent Aβ deposition in brain tissue and neurotoxicity (3). As a consequence, ApoE metabolism and function are recognized as both chemoprevention and therapeutic targets in AD (4). The possibility to tackle this target with natural products has stimulated a wealth of studies over the last decades (reviewed in Ref. 5). For example, the soy isoflavone genistein has demonstrated great potential to cure cognition and memory symptoms in a genetically-determined murine model of AD (6). Genistein was identified to produce a remarkable activity on PPARγ-dependent stimulation of ApoE expression (7).

Also α-tocopherol (α-TOH, Fig. 1), the most abundant form of vitamin E and the main fat-soluble antioxidant of human tissues (8), has long been considered a natural neuroprotectant and regulatory molecule of brain immune-inflammatory cells, *i.e.* glial that include astrocytes, oligodendrocytes, and microglia (9–11). Similarly to genistein, α-TOH is reported to influence the transcriptional activity of PPARγ (12, 13), as well as of PXR (14, 15), which may explain the reported influence of this vitamin on ApoE metabolism of the brain and other tissues (16, 17). Again, α-TOH was previously described to reduce Aβ levels and amyloid deposition in Tg2576 transgenic mice (18).

**Figure 1.**
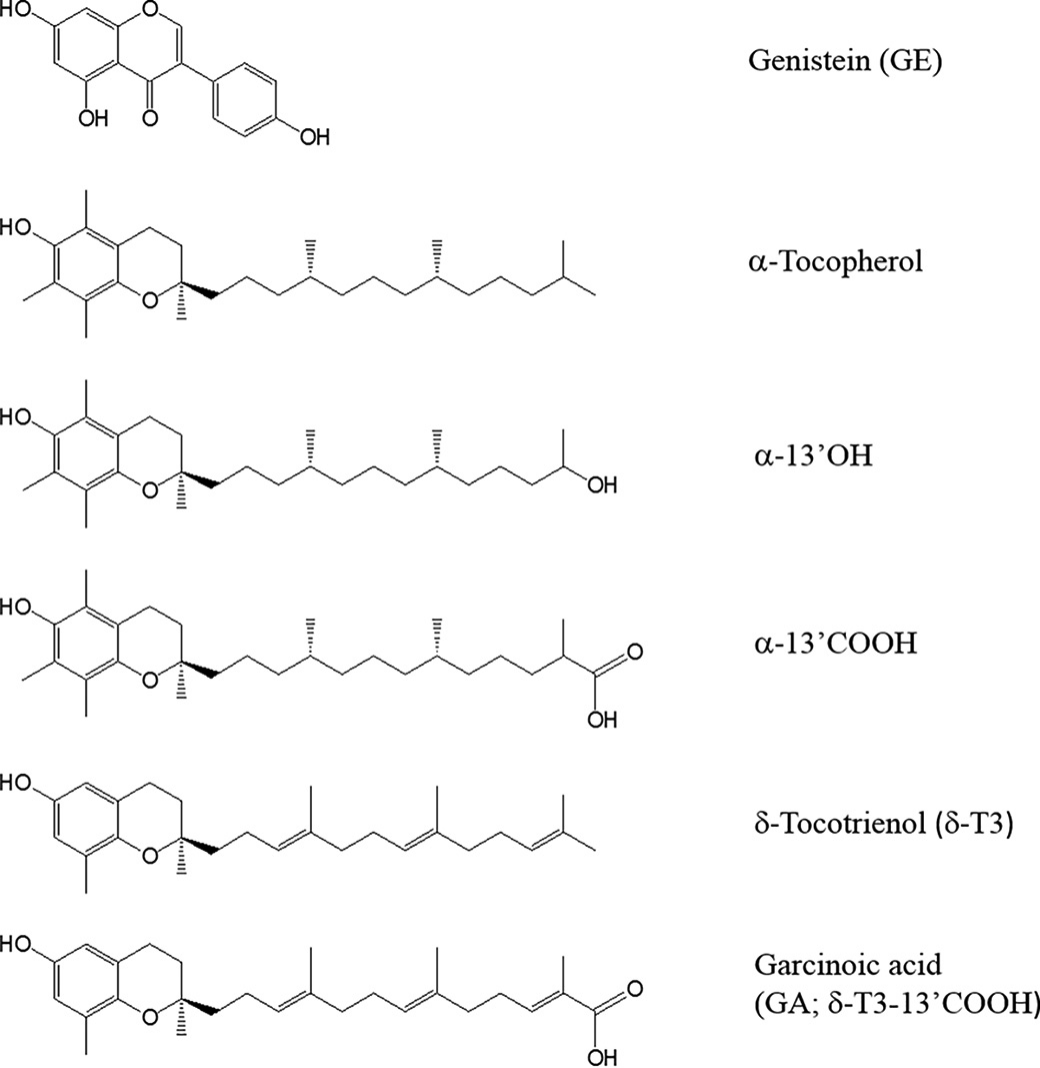
**Structures of test compounds.** GE, α-TOH, and its LCM α-13' OH, α-13'COOH, δ-T3, and its metabolite analog garcinoic acid (δT3-13' COOH).

Preliminary studies by some of us described a potent effect of long-chain metabolites (LCMs) of vitamin E (Fig. 1) as inducers of PPARγ expression in human hepatocytes (19). These LCMs included the CYP450-derived metabolites of α-TOH, *e.g.* α-13'OH and α-13'COOH, and garcinoic acid (GA or δ-13'COOH) (20), a natural analog of LCMs deriving from delta-tocotrienol (δ-T3) structure (Fig. 1). Recent evidence in literature also suggested a function of these LCMs (15), and especially of GA (21), as PXR agonists. This nuclear receptor is a central hub of xenobiotic metabolism (22) and its transcriptional role is proposed to control the CYP450-mediated metabolism of vitamin E, possibly by CYP4F2 and CYP3A4 isoenzyme regulation (14, 23, 24). Several studies also suggested a role of PXR in Aβ clearance through P-glycoprotein (P-GP or MDR1) dependent transport at the blood-brain barrier (25–27).

These effects of GA and other LCMs on nuclear receptor activity, along with the capability of vitamin E to cross the blood-brain barrier (28), are features in common with genistein, which may hold potential to prevent Aβ deposition and neurotoxicity. Therefore, in this study GA and a series of vitamin E analogs, were investigated for their effect on Aβ aggregation and metabolism in mouse cortical astrocytes. The properties of GA were also investigated in a transgenic mouse model that spontaneously accumulate amyloid plaques.

## Results

### In vitro studies

#### 

##### Cell-free assay of Aβ aggregation

**Figure 2.**
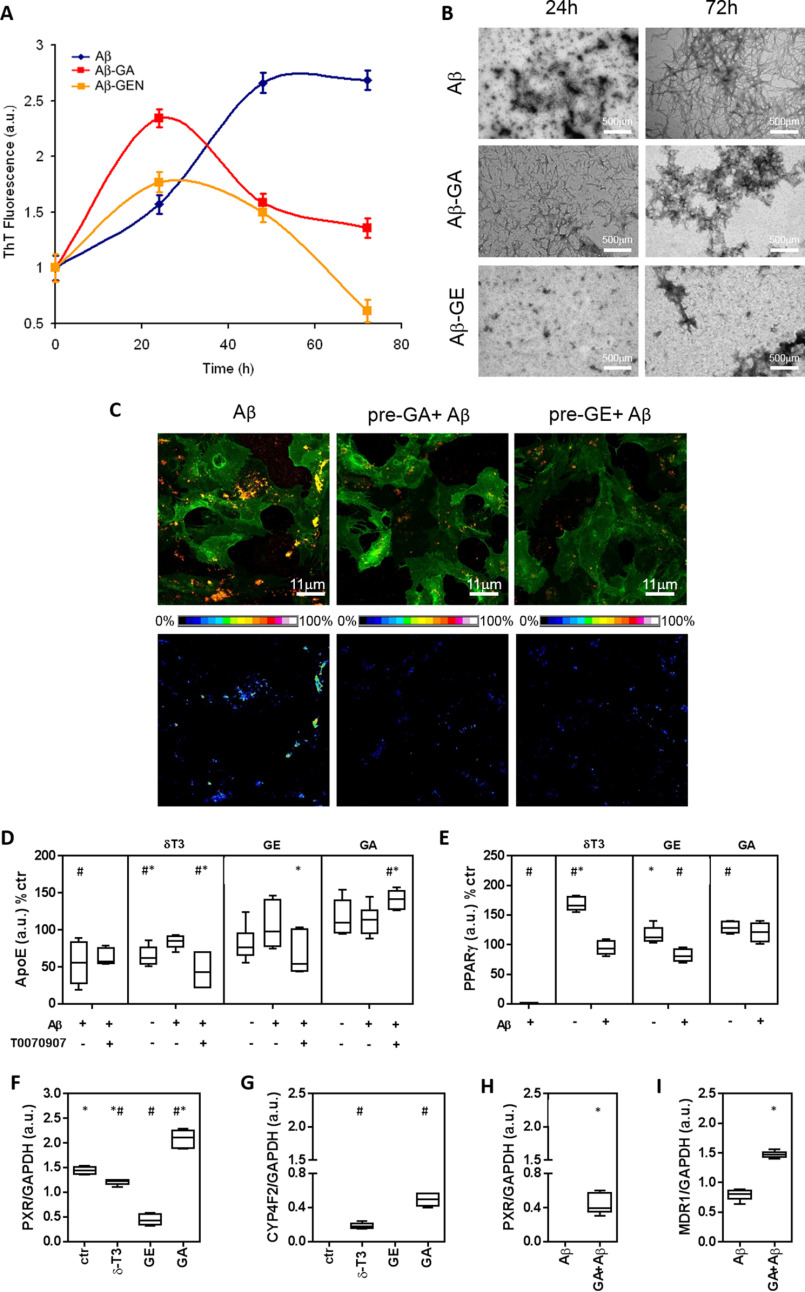
**Aβ aggregation and metabolism in mouse cortical astrocytes treated with genistein or garcinoic acid.** *A,* ThT fluorescent test was utilized to assess cross-β-sheet structure of Aβ(1–42) during formation of amyloid aggregates in cell-free experiments. Fluorescence was investigated for 72 h in the absence or in the presence of GE (5 μm) or GA (25 μm). *B,* Structural aspects of Aβ aggregation were investigated by transmission EM; *scale bars,* 500 μm. Aβ(1–42) aggregates on the plasma membrane of mouse astrocytes pre-treated with test molecules were assessed by immunofluorescence. *Scale bars*, 11 μm. *D–I*, immunoblot of extracellular ApoE and cellular levels of PPARγ, PXR, CYP4F2, and MDR1. Determinations were carried out in mouse cortical astrocytes pre-treated with GE (5 μm), GA (25 μm) or δ-T3 (2.5 μm), and then exposed to Aβ. In some experiments, the effect of the PPARγ activity inhibitor T0070907 was also investigated (*D*). Further details on cell treatments and determinations are reported under :Experimental procedures.” #, *p* < 0.05 *versus* Ctr test; *, *p* < 0.05 *versus* Aβ test.

The ThT fluorescence assay was utilized to investigate whether GA directly influences Aβ aggregation in cell-free experiments (Fig. 2*A*). Aβ42 fibrils formation showed a sigmoidal kinetics that reached a maximum of fluorescence after 48 h of incubation. In the presence of GA or GE, Aβ aggregation peaked at 24 h then decreasing progressively up to 72 h. TEM images (Fig. 2*B*) showed that GA treatment causes the formation of short protofibrils after 24 h, followed by the presence of amorphous ThT negative material at 72 h. GE also inhibited the development of mature fibrils after 72 h.

##### Aβ aggregation and metabolism in mouse astrocytes

First, acute toxicity in mouse astrocytes was excluded for all the test compounds (GE, α-TOH, α-LCMs, GA, and δ-T3) investigated either separate (Fig. S1*A* and Table S1) or combined with Aβ treatment (Fig. S1*B*, Table S1). In these tests cell viability was assessed by trypan blue dye exclusion (Fig. S1, *A* and *B*), MTT and CCK-8 assay (Table S1). Immunolocalization of Aβ1-42 aggregates demonstrated that the pre-treatment with GA and GE reduces the number and dimension of Aβ oligomers that interact with the plasmalemma of murine astrocytes (Fig. 2*C*).

##### ApoE efflux and nuclear receptor function

As a major finding in this study, Aβ was found to significantly reduce the capability of astrocytes to release ApoE in the extracellular medium (Fig. 2*D*) and this negative effect of Aβ was completely prevented by the pre-treatment with GA as well as of genistein (Fig. 2*D*) and α-13'OH, but not α-13'COOH (Fig. S2). In the absence of Aβ, GA stimulated ApoE efflux (Fig. 2*D*), whereas a trend toward a decreased efflux was observed in the case of α-13'COOH and α-13'OH (Fig. S2).

The PPARγ activity inhibitor T0070907 was utilized to verify the functional contribution of this nuclear receptor in the response of ApoE efflux to GA and other compounds, a role that has already been described for genistein (2, 16). The treatment with α-13'COOH was excluded from this experiment because of its negative effect on ApoE efflux after Aβ treatment (Fig. 2*D*). T0070907 hampered part of the stimulation effect that GE and α-13'OH produced on ApoE efflux. This effect was not observed on the efflux response to GA that rather increased in the presence of this PPARγ inhibitor (Fig. 2*D*). This finding reveals a PPARγ-independent stimulation effect of GA on ApoE efflux in mouse cortical astrocytes exposed to Aβ. GA increased PPARγ protein expression (Fig. 2*E*) restoring its levels that, analogously to ApoE efflux (Fig. 2*D*), were decreased after Aβ treatment. With the exception of α-13'OH, pretreatments with all the other vitamin E molecules restored the expression of this NR with remarkable increases observed in the case of α-13'COOH and δ-T3 (Fig. 2*E* and Fig. S2*E*).

Because the activity of GA appears to be at least in part independent from PPARγ activity, another molecular target of this plant metabolite that may sustain ApoE transport and Aβ clearance through P-GP or MDR1 up-regulation (25–27, 29), is PXR (15, 21). GA was confirmed to be a potent activator of PXR protein expression also in mouse astrocytes (Fig. 2, *F* and *H*) and, together with δ-T3, it significantly stimulates CYP4F2 (Fig. 2*G*) and MDR1 (Fig. 2*I*) expression. These are PXR reporter genes not affected by the PPARγ-activating compound genistein. Similarly to ApoE and PPARγ, PXR expression was also reduced in mouse astrocytes by the treatment with Aβ, and GA pre-treatment restored the levels of this nuclear receptor (Fig. 2*H*).

### GA availability, modulation of target genes, and aβ metabolism in mouse brain

**Figure 3.**
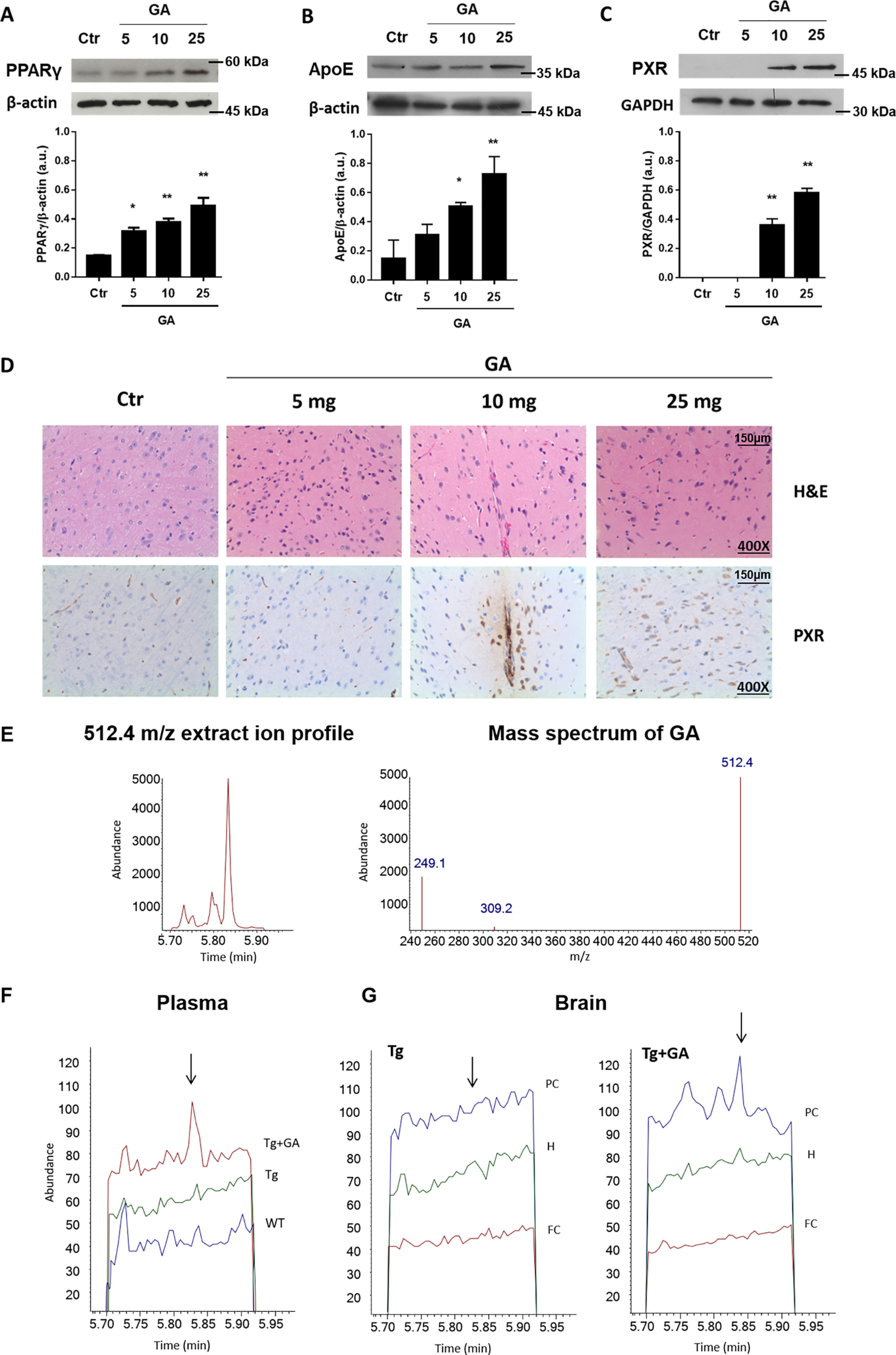
**Brain target engagement and bioavailability of GA.** Immunoblot was utilized to assess PPARγ (*A*), PXR (*B*), and ApoE (*C*) expression in brain of C57Bl/6 mice treated with different concentrations of GA. The brain PXR expression of these animals was also verified by IHC analysis (*D*). GC-MS was utilized to assess the availability of GA (mass spectrum and ion chromatogram at *m*/*z* 512.4 for GA detection are shown in *E* to plasma (*F*) and brain tissue (*G*) of WT, Tg and Tg+GA mice. Brain samples were collected in three different areas: parietal cortex (*PC*), hippocampus (*H*), and frontal cortex (*FC*). Data were as mean ± S.D. of three experiments. *, *p* < 0.05 *versus* Ctr test; **, *p* < 0.01 *versus* Ctr test.

Bioavailability and target engagement of GA was investigated in mouse brain. In C57Bl/6 mice administered with a single bolus of GA, the concentration-dependent response of PPARγ, PXR, and ApoE expression was demonstrated by immunoblot (Fig. 3, *A*–*C*), and PXR up-regulation was also confirmed by IHC with some astrocytes showing cytoplasmic localization of the receptor at the higher dose of GA investigated in this study (25 mg) (Fig. 3*D*).

Bioavailability of GA in Tg mice was assessed in plasma and brain samples by GC-MS analysis (Fig. 3, *F* and *G*). Semiquantitative results of brain samples were compatible with GA concentrations in parietal cortex (PC) and hippocampus (H) of Tg mice treated with GA (Tg + GA) between 0.8 and 10.3 ppm (ng/mg of proteins) (Fig. 3*G*, *right panel*).

**Figure 4.**
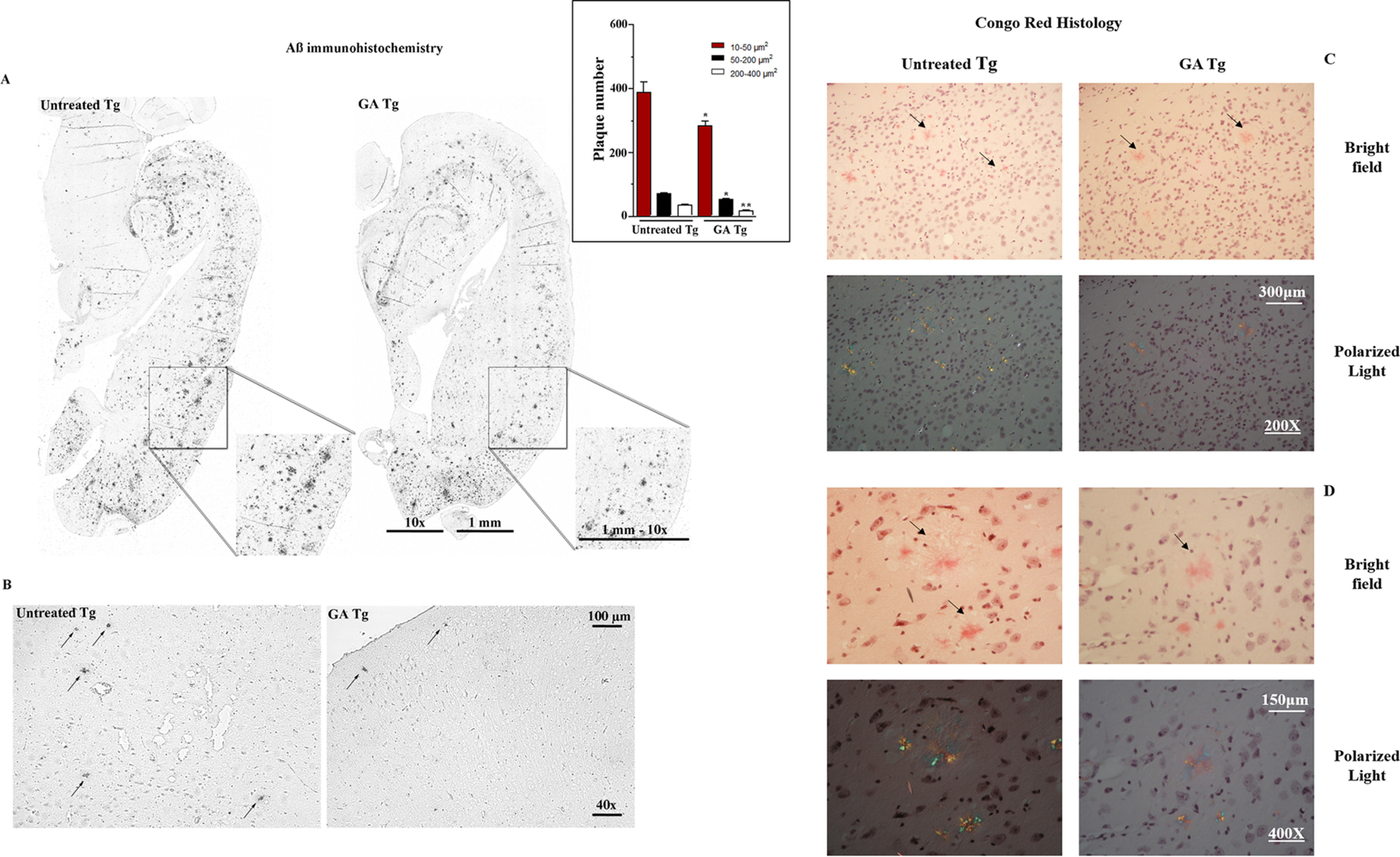
**Effect of GA on Α**β **aggregate formation in the brain of TgCRND8 mice.** IHC analysis of pyroglutamate-3 Aβ (pE3 -Aβ) (*A* and *B*) and Congo Red histology (*C* and *D*) of entire hemibrain sections were performed in TgCRND8 mice after 10-day oral administration of GA (200 mg/kg; Tg+GA group) or vehicle (olive oil; Tg group). Control images of Congo Red staining are reported under Fig. S5. Other details on these animal experiments are reported under “Experimental Procedures.” *Insert* to *panel A*: bar chart of Aβ plaque number in the entire hemisphere of 14-15-month–old Tg mice; plaque number was determined automatically on digitized images using the CellSens Dimension software (Olympus, Germany); **, *p*< 0.005 and *, p< 0.05 *versus* respective area size of untreated Tg.

IHC data demonstrated that GA prevents the formation and reduces the size and morphological complexity of Aβ plaques throughout the brain of Tg mice, namely the entire cortical mantle and subcortical regions (Fig. 4, *A* and *B*). Quantitative analysis of the pE3 -Aβ plaque number in 14-15-month–old Tg mice demonstrated a significant reduction in the number of all size amyloid plaques by GA treatment (*inset* to Fig. 4*A*). These findings were also confirmed by Congo Red staining of brain samples (Fig. 4, *C* and *D*); in these experiments the decreased accumulation of Aβ deposits was particularly evident in oldest Tg mice (14 to 15-month–old) that accumulate a much severe form of Aβ deposits (*upper panels*) compared with youngest (5-month–old Tg mice, lower panel). Representative control images of Congo Red histology are shown in Fig. S5.

ApoE protein expression assessed by immunoblot (Fig. S3*B*) significantly increased in the hippocampus of Tg mice treated with GA. Similarly, GA stimulated MDR1 expression in the hippocampus (Fig. S3, *C* and *D*) and parietal cortex (not shown).

Immunohistochemistry data did not show a marked effect of GA treatment on PXR expression in brain tissue of Tg mice (Fig. S4*A*). PXR is widely present in the brain tissue Tg mice, but in lower amount compared with the liver (Fig. S4, *A* versus *B*). In hepatocytes of Tg mice treated with GA, slightly increased levels of PXR were observed (Fig. S4*B*). According to Ref. 30, PXR is highly expressed in the gut and GA treatment stimulates its expression, mainly in enterocyte crypts (Fig. S4*C*).

## Discussion

In this study, GA, the natural analog of δ-T3-13'COOH, has been evaluated for the first time in an *in vitro* model of Aβ aggregation and cytotoxicity (31), and *in vivo* in TgCRND8 mice, a transgenic model of Aβ deposition (32). Aβ aggregation, and the gene response associated with the cellular metabolism of this peptide, have been evaluated.

GA showed a good safety profile (absence of major cytotoxic effects at micromole levels) and was the most efficient between the vitamin E analog investigated in this study to decrease Aβ accumulation in mouse astrocytes. Mechanistic investigation indicates that similarly to genistein, GA interferes with the aggregation properties of Aβ and it is effective in stimulating ApoE efflux and MDR1 transporter expression, which are important to sustain the cellular clearance of the peptide. In fact, thereby preventing its interaction with the plasmalemma (Fig. 2), which are important prerequisites to lessen its intrinsic cytotoxicity (31). This detoxification response to GA could be explained by the marked up-regulation of PPARγ and PXR nuclear receptors, the expression of which is impaired by the treatment with Aβ. The fact that GA efficiently prevented the defect of these receptors, restoring MDR1 levels and the efflux of ApoE, may thus suggest a chemoprotection function for this natural compound.

The PPARγ activation response of GA confirms and extends the preliminarily findings obtained in human hepatocytes (19), indicating similarities with the therapeutic mechanism of genistein. Our data demonstrate that this soy isoflavone is a potent agonist of the PPARγ-induced efflux of ApoE in Aβ-treated astrocytes, possibly more potent than GA. This PPARγ-mediated response to genistein was already indicated to explain its therapeutic potential in genetically-determined AD mice (6). Moreover, it may have effects on both the phagocytosis of Aβ by microglial cells (3) and its clearance by the activation of brain target genes involved in the reverse cholesterol transport, such as ApoE and ABCA1 (33).

Pharmacological inhibition experiments, however, suggested that the ApoE efflux response to GA, but not to GE or other vitamin E molecules, such as δ-T3 and α-13'OH, is at least in part independent from PPARγ activity. Nuclear receptors alternative to PPARγ, that may produce similar or even more potent effects on ApoE transcription and efflux, include PXR. In fact, this receptor is reported to influence ApoE transport and Aβ clearance through P-glycoprotein (P-GP or MDR1) up-regulation (25–27, 29).

Our findings demonstrate for the first time an agonistic effect of GA on brain PXR. Upon treatment, PXR increased its own expression and that of the reporter gene MDR1, a response that was already described for LCMs and δT3 in intestinal cells (15). GA up-regulated MDR1 expression not only in mouse astrocytes, but also *in vivo* in the brain of WT and Tg mice. Moreover, we demonstrate that the treatment with GA, but not genistein, stimulates the expression of another PXR-regulated gene with proposed activity in the ω-oxidation of vitamin E side chain, *e.g.* CYP4F2 (34). These results are in line with recent experimental evidence that demonstrates a selective PXR agonist activity of GA, which is specific for this vitamin E metabolite (21). Consistent biochemical and functional evidence support these findings including the co-crystallization of GA with the LBD of PXR nuclear receptor, thermodynamic data, target engagement and the transcriptional response of different tissues that include gut and liver tissues in which PXR is abundantly expressed and important for its detoxification function (21). The response of intestinal and hepatic PXR to GA treatment is confirmed in the present study and it is described for first time to extend to some areas of the brain (Fig. 3), which may help to explain the improved Aβ deposition phenotype of TgCRND8 mice (Fig. 4).

However, the absence of a consensus sequence in the promoter region of the *APOE* gene specific for this nuclear receptor and its dimeric complex with RXR (assessed in Refs. 35 and 36), may lead to exclude a direct transcriptional effect of PXR on *APOE* gene. Considering indirect effects, PXR-dependent genes involved in this response may include *ABCA1* and/or *MDR1* gene regulation with important role in preventing Aβ deposition (25–27, 29). At the same time, target engagement experiments in WT mice (Fig. 3) demonstrate that PXR and PPARγ are both activated with a similar concentration-dependent response to GA in the brain. This may lead to hypothesize converging mechanisms of transcriptional regulation by NR on *APOE* and other genes with key role in Aβ metabolism that are worth investigating further.

Cellular experiments in study further emphasizes the concept that different vitamin E derivatives have different gene regulatory functions (37, 38). Compared with δ-T3, the use of GA may directly modulate ApoE activity avoiding the need for an efficient bioavailability and metabolism of δ-T3 to form δ-T3-13'COOH in the brain. Important enough, GA shows much lower cytotoxicity compared with δ-T3, thus allowing treatments with much higher doses; furthermore, the more hydrophilic properties of GA may favor compound permeability through the blood-brain barrier. The fact that GA is available to the mouse brain and, along with PPARγ, it up-regulates PXR expression of this tissue, suggests other pharmacological applications, including anxiety-like behavior and recognition memory (39), and blood barrier function modulation (40) that are all PXR-dependent processes. Other therapeutic effects of GA could be anticipated in lipid and metabolic disorders associated with higher demand of ApoE activity or unfavorable *APOE* haplotypes (41, 42).

In conclusion, in the present study GA was demonstrated to have compound-specific effects useful to prevent Aβ accumulation in the mouse brain, being capable at the same time of 1) interfering with Aβ polymerization, 2) stimulating the ApoE and MDR1 detoxification system of mouse cortical astrocytes and the brain tissue, and 3) reducing Aβ deposition in the brain of TgCRND8 mice, an animal model of AD. Pharmacological and target engagement data suggest that the PXR and PPARγ agonist properties of GA could help to explain a better cellular clearance of Aβ by the increased expression and activity of ApoE and MDR1 transporter. Altogether these findings indicate that GA holds potential in chemoprevention and therapy of AD that is worth of further investigation.

## Experimental Procedures

### Thioflavin-T (ThT) and transmission EM of Aβ1-42 aggregates

ThT was utilized as a fluorescent probe to the specifically bound cross-β-sheet structure of amyloid. Formation of Aβ(1-42) aggregates was investigated for different times between 0  and 72 h in the absence or presence of GE (5 μm) or GA (25 μm). The aggregates were diluted to 15 μm monomeric peptide concentration with 20 mm phosphate buffer, pH 7.4, at 25 °C, and then were supplemented with ThT (20 μm final concentration). The samples were transferred into 96-well–plate, and ThT fluorescence (450 excitation/482 emission) was recorded using a Biotek Synergy 1H plate reader. For TEM analysis, 5-μl aliquots of Aβ(1-42) aggregated were withdrawn at 24 and 72 h, and loaded onto a formvar/carbon-coated 400 mesh nickel grids (Agar Scientific, Stansted, UK) and negatively stained with 2.0% (w/v) uranyl acetate (Sigma-Aldrich). The grid was air-dried and examined using a JEM 1010 transmission electron microscope at 80 kV excitation voltage.

### Cell culture conditions and treatments

Mouse cortical astrocytes were cultured from dissected cerebral cortices of neonatal C57BL/6J mice. 3 to 4 brain samples were utilized to seed the cell suspension in a 150-cm^2^ culture flask (NUNC, Roskilde, Denmark). The cells were maintained at 37 °C in a humidified incubator with 5% CO_2_ atmosphere, utilizing Dulbecco's modified Eagle's medium/F-12 culture medium (1% l-glutamine) supplemented with 10% fetal bovine serum (Thermo Fisher Scientific), 62.5 µg/ml of penicillin and 100 µg/ml of streptomycin (Sigma-Aldrich)_._ At confluence, the astrocytes were seeded in 25-cm^2^ culture flasks at a density of 5 × 10^5^ cells per flask and were maintained in culture for 5 to 6 days (80–90% confluence) before utilization. Treatments were carried out to assess GA and other test compounds (Fig. 1) in chemoprevention mode with the following protocol:

#### 

##### Pre-treatment step

The cells were exposed for 24 h to vehicle (0.001% (v/v) ethanol) or test compounds: genistein (5 μm; Sigma-Aldrich), α-TOH, α-13'-OH, and α-13'-COOH LCMs, GA (25 μm final concentration each; prepared according to Refs. 21 and 43), and δ-T3 (2.5 μm, Cayman).

##### Treatment step

After washing, the cells were exposed for 24 h to 5 μm Aβ(1-42) (GenScript) in fresh medium. These conditions of treatment (time of exposure and concentration of Aβ) were selected to maintain the reduction of cell viability < 20% and thus to perform chemoprevention experiments with a sufficient number of viable cells (Table S1). Culture media were collected after pre-treatment and treatment steps for ApoE determination. In some experiments, the pre-treatment step was performed in the presence of the PPARγ activity inhibitor T0070907 (10 nm in EtOH, Sigma Aldrich) (44).

### Cell viability

#### 

##### Trypan blue exclusion assay

After treatment, the cells were washed in PBS and then detached with Trypsin-EDTA for collection by centrifugation and resuspension in 2 ml of culture medium containing 0.2% (w/v) trypan blue. Trypan blue-positive cells and total cells were counted by a single operator under a light microscope in four (1 × 1 mm) fields of a Neubauer chamber.

##### MTT assay

The MTT reagent (3-(4,5-dimethylthiazol-2-yl)-2,5-diphenyltetrazolium bromide; Sigma-Aldrich) was added to astrocytes seeded in 96-well–plates (2 × 10^3^ cell/well) at 0.5 mg/ml final concentration. After a 3-h incubation step (37 °C), formazan crystals formed by the reducing activity of mitochondrial dehydrogenases on MTT were dissolved with 100 μl of solubilization solution and absorbance was measured at 570 nm in a microplate reader. Nonviable cells are unable to reduce the MTT dye.

##### CCK-8 assay

10 μl of CCK-8 reagent (Cell Counting Kit-8, Dojindo Molecular Technologies, Inc.) was added to each well seeded with 2.5 × 10^3^ astrocytes. After incubation (2 h), the absorbance was recorded at 450 nm wavelength using a microplate reader (Becton Dickinson).

### Immunofluorescence assay

Subconfluent astrocytes grown on glass coverslips were pre-treated with GE (5 μm) or GA (25 μm) for 24 h and then incubated with Aβ 5 μm for the following 24 h. The oligomers and the cell membrane were stained with rabbit anti-Aβ(1-42) polyclonal antibody followed by the Alexa Fluor 568-conjugated secondary antibodies (red channel) and the Alexa 488-toxin cholera (staining GM1 membrane; green channel), respectively. Cell fluorescence was imaged using a confocal Leica TCS SP5 scanning microscope (Leica, Mannheim, Germany) equipped with a HeNe/Ar laser source for fluorescence measurements. The observations were performed using a Leica Plan 7 Apo × 63 oil immersion objective. FRET analysis was performed by adopting the FRET sensitized emission method, as previously reported (45).

### Animal experiments

Blood and brain tissue availability of oral GA was preliminarily investigated in 8-week–old male C57Bl/6 mice (18–22 g; Charles River Laboratories International, Inc., Wilmington, MA, USA). All animal procedures were approved by the Animal Ethics Committee of the University of Perugia and were performed in accordance with the Italian code for care. Mice were randomly assigned to receive an oral dose of 5, 10, or 25 mg of GA (*n* = 3/dose cohort) or vehicle (olive oil, *n* = 3). At the end of treatment, the animals were anesthetized and sacrificed according to institutional guidelines, and blood and brain tissue samples were collected for histology and biochemistry assays. Grouping of animals and quantifications were blinded for the responsible researchers throughout the study.

To investigate the effect of GA on Aß deposits, 5-month and 14-15-month–old TgCRND8 (Tg) mice (*n* = 3) were used. Five-month–old WT (WT) (*n* = 2) mice were also utilized as controls. Tg mice harboring a double-mutant gene of APP695 (32) and WT control littermates were used in accordance with the principles of the Basel Declaration and the Working document on genetically altered animals - CORRIGENDUM of 24 January 2013, of the National Competent Authorities for the implementation of Directive 2010/63/EU on the protection of animals used for scientific purposes. According to the Italian Regulation (D.Lvo 26/2014), the protocol was revised and approved by the Animal Welfare Body of the University of Florence and licensed by the Italian Competent Authority (Italian Ministry of Health, license number 71/2017-PR). Animals were housed in macrolon cages with food and water *ad libitum* and maintained on a 12-h light/dark cycle at 23 °C. All efforts were made to minimize the number of animals used and their suffering.

Tg mice were orally administered with GA (200 mg/kg; Tg + GA group) or vehicle (olive oil; Tg group) for 10 days. The animals were weighed once a day, and the bodyweight recorded and the dose to be orally administered was recalculated according to the body weight. After completing the treatments, blood samples were collected into EDTA-coated tubes, then the animals were sacrificed by cervical dislocation and brains were quickly dissected on dry ice; frontal and parietal cortex and hippocampal samples were stored at −80 °C until use. Liver and gut specimens were also collected and fixed, as well as hemibrain samples, with 4% ice-cold paraformaldehyde in 0.1 m PBS, pH 7.2, for 24 h at 4 °C, and histochemical and immunohistochemical analyses were performed on 4 μm paraffin-embedded sections.

### GC-MS analysis of GA

Approximately 33 mg of homogenized brain tissues (parietal cortex, frontal cortex, and hippocampus) and 150 μl of plasma were utilized to determine GA availability in TgCRND8 mice. Samples were mixed with 1 ml of sodium acetate buffer, pH 5.0, and incubated overnight at 37 °C with 40 µl of an enzymatic mixture containing β-glucuronidase (≥100,000 units/ml) and *Helix pomatia* type H2 sulfatase (≤7500 units/ml) (Sigma-Aldrich Chemie GmbH, Steinheim, Germany). Salting out extraction of hydrolyzed samples was carried out by QuEChERS (Phenomenex, Italy) utilizing acetonitrile, anhydrous magnesium (4 g), sodium chloride and sodium citrate (1 g each), sodium citrate dibasic sesquihydrate (0.5 g), followed by a dispersive solid-phase cleanup step with anhydrous magnesium (900 mg), and 150 mg each of PSA and C18 (Phenomenex, Italy). Further purification of the extracted samples was carried by silica gel TLC. The concentrated extracts were methylated for 30 min in 3 n methanolic HCl and silylation was carried out utilizing N,O-bis(trimethylsilyl)trifluoroacetamide + 1% trimethylsilyl chloride/pyridine at 65 and 60 °C, respectively. Samples were analyzed using an Agilent Technologies GC-MA work station consisting of a MSD 5975C Triple Axis Detector coupled with a gas chromatograph (GC 7890A) equipped with an VF-5 ms capillary column (15 m × 0.15 mm inner diameter, 0.15 μm film thickness). GA analysis was calibrated utilizing an authentic standard prepared according to Refs. 21 and 43.

### Immunoblot of astrocytes and brain tissue proteins

To extract astrocytes proteins, the cells were incubated for 40 min at 4 °C in lysis buffer (Cell Signaling Technologies) supplemented with protease and phosphatase inhibitor mixture (Pierce, Thermo Fisher Scientific), and fresh 1 mm phenylmethylsulfonyl fluoride (Sigma-Aldrich, MO, USA). After incubation, the samples were centrifuged (14,000 rpm for 30 min at 4 °C), and the supernatants and pellets were collected for immunoblot analysis. Cell culture media of astrocytes were also collected and processed for protein extraction to measure ApoE efflux.

Brain homogenization for protein extraction (∼200 mg of tissue) was carried out in protein extraction buffer (Trizma (Tris base), 10 mm Nonidet P-40, KCl 154 mm, and fresh phenylmethylsulfonyl fluoride, pH 7.6) with freshly added protease inhibitors (DTT, leupeptin, and aprotinin) using mortar-pestle and sonication methods. After centrifugation (27,000 × *g*, 20 min), the supernatant was collected for immunoblot. Membrane proteins were separated utilizing a Cell Fractionation Kit (#9038, Cell Signaling Technology Inc.) according to manufacturer's instructions. Total proteins of cell lysates or brain homogenates were quantified by BCA assay (Thermo Fisher Scientific).

Immunoblot of ApoE, was performed on both the cell culture media and cellular extracts by protein separation on 10% SDS-PAGE and subsequent electroblotting to a polyvinylidene difluoride membrane. After blocking with 5% nonfat milk, the membrane was incubated with an anti-Lipoprotein E antibody (ab83115; Abcam). For the other antigens, proteins (20 µg) were resolved by 12% SDS-PAGE and transferred to a nitrocellulose membrane (Thermo Fisher Scientific). After blocking with 5% nonfat milk, the blots were incubated with primary antibodies (anti-ApoE (1:1000, Abcam), anti-CYP4F2 (1:500, Santa Cruz Biotechnology), anti-PPAR-γ (1:1000, Cell Signaling Technologies), anti-PXR (1:200 dilution; Santa Cruz Biotechnology, Inc.), anti-MDR1 (1:1000, Cell Signaling Technologies) anti-α-tubulin control (Abcam-ab4074), anti-GAPDH (1:1000, Cell Signalling Technologies), anti-β-actin (1:1000, Cell Signalling Technologies), and anti-β-catenin (1:2000, BD Transduction Laboratories™)), and then with an horseradish peroxidase-conjugated secondary antibody (1:2000, Cell Signaling Technologies). Total protein normalization was performed using Ponceau S staining. Band detection was carried out by ECL-plus solution (Pierce, Thermo Fisher Scientific) according to the manufacturer's instructions. Images of blotted membranes stained with Ponceau and films were captured by “Imagequant LAS4000” (GE-Healthcare Bio-Sciences) and analyzed with “Image J” software.

### IHC and Congo Red histochemistry

Brain, liver, and gut tissue specimens were formalin-fixed and paraffin-embedded. Four micrometer sections were mounted on polarized glass slides. One slide from each case was stained with hematoxylin and eosin (Sigma-Aldrich). Immunohistochemical analysis of PXR (polyclonal IgG, dilution 1:200; Abcam) was performed using Bond III automated slide preparation system and Polymer Refine antibody detection system (Vision BioSystems, Buffalo Grove, IL, USA). Diaminobenzidine was used as chromogen. The sections were then counterstained with hematoxylin and mounted with Digital Picture Exchange.

For Cong Red staining 8-μm sections were cut from formalin-fixed and paraffin-embedded mouse brain tissue and mounted on glass slides. Staining was performed using Congo Red Stain Kit (DAKO) on an automated slide preparation Dako's Artisan Staining system. Sections were then counterstained with hematoxylin and mounted according to manufacturer's instruction. Samples were evaluated using both standard and polarized light microscopy. Aβ plaque burden was analyzed in brain slices stained with the anti-N3pE antibody (1:200 diluted, IBL international, Hamburg, Germany) (46), which recognizes the pyroglutamate-3 Aβ (pE3 -Aβ) and was digitized and acquired with an Olympus BX63 microscope equipped with the *CellSens Dimension* software (Olympus, Germany). The number of pE3-Aβ plaques of different sizes was determined in 14-15-month–old Tg mice staining 1 entire hemisphere section of each mouse brain.

### Statistics

Data were as mean ± S.D. and statistical significance was evaluated by one-way analysis of variance followed by Bonferroni post hoc test, utilizing the statistics package of GraphPad Prism version 5.0 (San Diego, CA, USA); Student's *t* test was used to compare differences between pE3-Aβ plaque data. A probability of error <0.05 was considered significant.

## Data availability

All data are contained within the article.

## Supplementary Material

Supporting Information
