# Supplementary material for: Garcinoic acid prevents β-amyloid (Aβ) deposition in the mouse brain
Source: J Biol Chem. 2020 Jul 2;295(33):11866–76. doi: 10.1074/jbc.RA120.013303 (PMC7450134; doi:10.1074/jbc.RA120.013303)
Supplement: Supporting Information [file supp_295_33_11866__index.html]

Garcinoic acid prevents β-amyloid (Aβ) deposition in the mouse brain — Garcinoic acid reduces β-amyloid deposition in the mouse brain — Garcinoic acid prevents β-amyloid (Aβ) deposition in the mouse brain — Garcinoic acid reduces β-amyloid deposition in the mouse brain — Supporting Information 

# Garcinoic acid prevents β-amyloid (Aβ) deposition in the mouse brain

## Supporting Information

- Supporting Information JBC-2020-013303R1 (to be published online) - Revised documents: Supporting Table S1 and Supporting Figures S1-S5
