# Supplementary material for: Garcinoic acid prevents β-amyloid (Aβ) deposition in the mouse brain
Source: J Biol Chem. 2020 Jul 2;295(33):11866–76. doi: 10.1074/jbc.RA120.013303 (PMC7450134; doi:10.1074/jbc.RA120.013303)
Supplement: Supporting Information [file supp_RA120.013303_159234_3_supp_557501_qcr199.pdf]

Supplementary Table S1. Cell viability results of pre-treatment (upper chart) and treatment (lower chart) phases of the in vitro study on mouse cortical astrocytes as determined with MTT and CCK-8 test.

	**MTT Assay**			**CCK-8**		
**Cell vitality**	**Mean**	**SD**	**% Ctr**	**Mean**	**SD**	**% Ctr**
Ctr	0.27	0.01	100	0.47	0.02	100
α-TOH	0.25	0.01	92.3	0.68	0.05	142.6
α-13'OH	0.26	0.01	104.2	0.60	0.01	126.6
α-13'COOH	0.26	0.01	101.0	0.59	0.04	123.0
δ-T3	0.25	0.01	94.4	0.58	0.01	121.8
GA	0.24	0.01	95.4	0.55	0.08	116.3
GE	0.26	0.01	113.3	0.51	0.01	107.1

	**MTT Assay**			**CCK-8**		
**Cell vitality**	**Mean**	**SD**	**% Ctr**	**Mean**	**SD**	**% Ctr**
Ctr	0.29	0.01	100	0.59	0.06	100
Aβ	0.27	0.01	90.8	0.47	0.03	78.8
α-TOH	0.31	0.01	121.3	0.57	0.04	95.7
α-13'OH	0.31	0.01	100.5	0.51	0.01	86.3
α-13'COOH	0.32	0.05	107.7	0.53	0.05	90.0
δ-T3	0.29	0.01	87.7	0.54	0.05	90.1
GA	0.30	0.01	101.9	0.52	0.01	88.3
GE	0.29	0.01	96.5	0.50	0.05	84.6

Results are presented as mean and standard deviations of experiments run in triplicate.

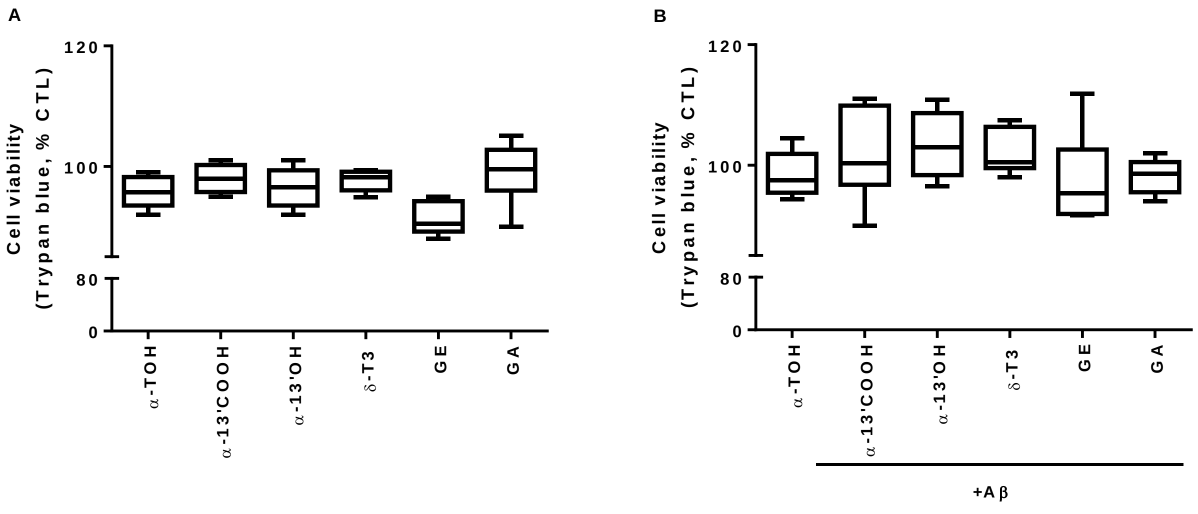

Suppl. Figure S1. Cell viability of mouse cortical astrocytes pre-treated with genistein or vitamin E compounds and exposed to Aβ. Mouse cortical astrocytes were pre-treated with the test compounds for 24 hrs and then were exposed to 5 µM Aβ for other 24 hrs. Along with genistein (5 µM), the test compounds included the following vitamin E molecules (25 µM final concentration in the cell culture medium, if not otherwise specified): α-TOH, α-13'OH, α-13'COOH, and GA. δ-T3 was utilized at 2.5 µM final concentration. Data were as means ± SD of three independent experiments. # $p<0.05$ vs Ctr test.

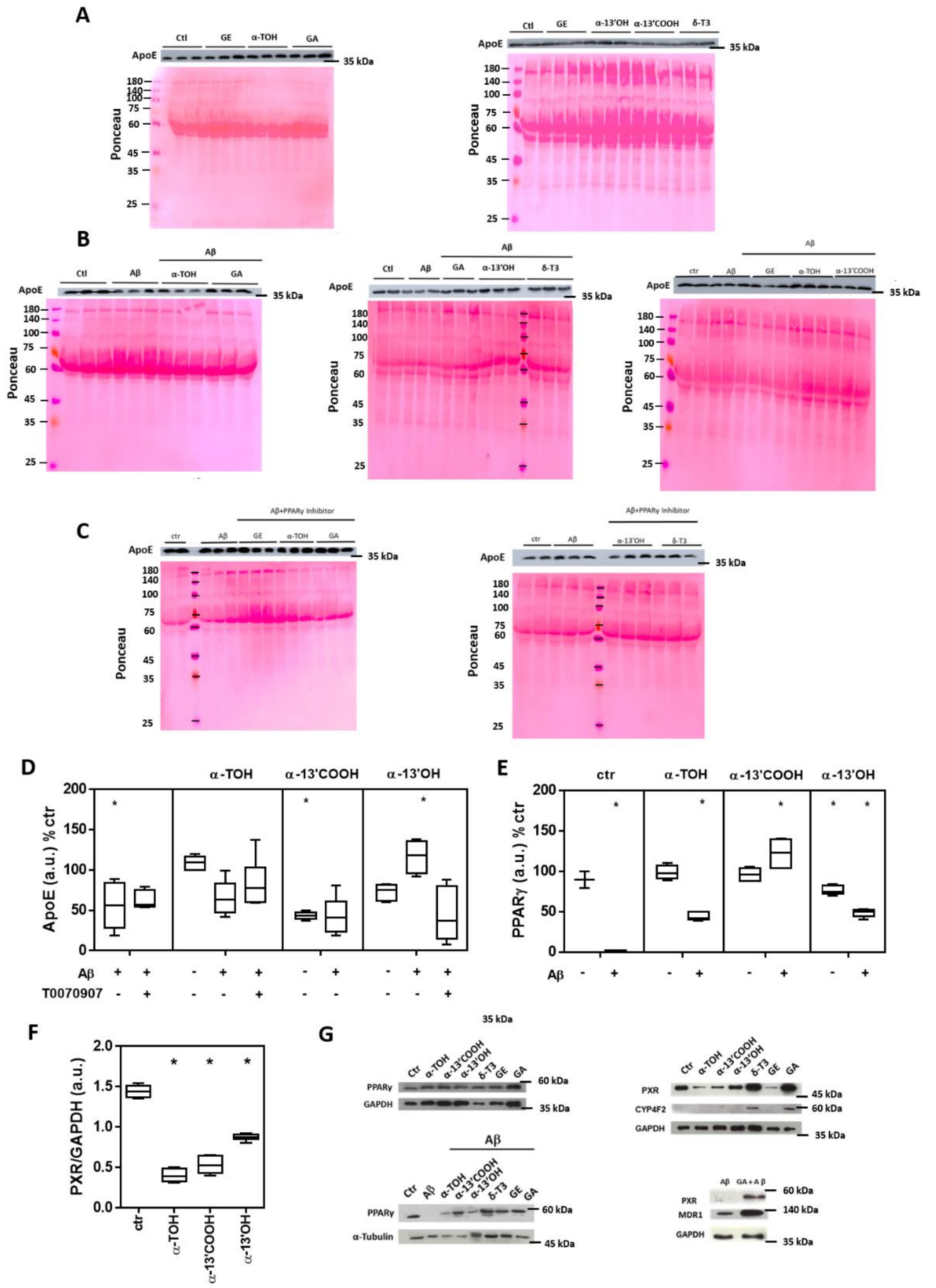

Suppl. Figure S2. Immunoblot of extracellular ApoE and cellular levels of PPARγ, PXR, CYP4F2 and MDR1 in mouse cortical astrocytes pre-treated with genistein or vitamin E compounds and exposed to Aβ. Final concentrations of the test compounds were: GE = 5 µM, δ-T3 = 2.5 µM and GA or other vitamin E molecules = 25 µM. Further details on cell treatments and determinations are reported in the Experimental procedures. In some experiments, the effect of the PPARγ activity inhibitor T0070907 was also investigated (panel D). #p< 0.05 vs Ctr test, ∗p<0.05 vs Aβ test.

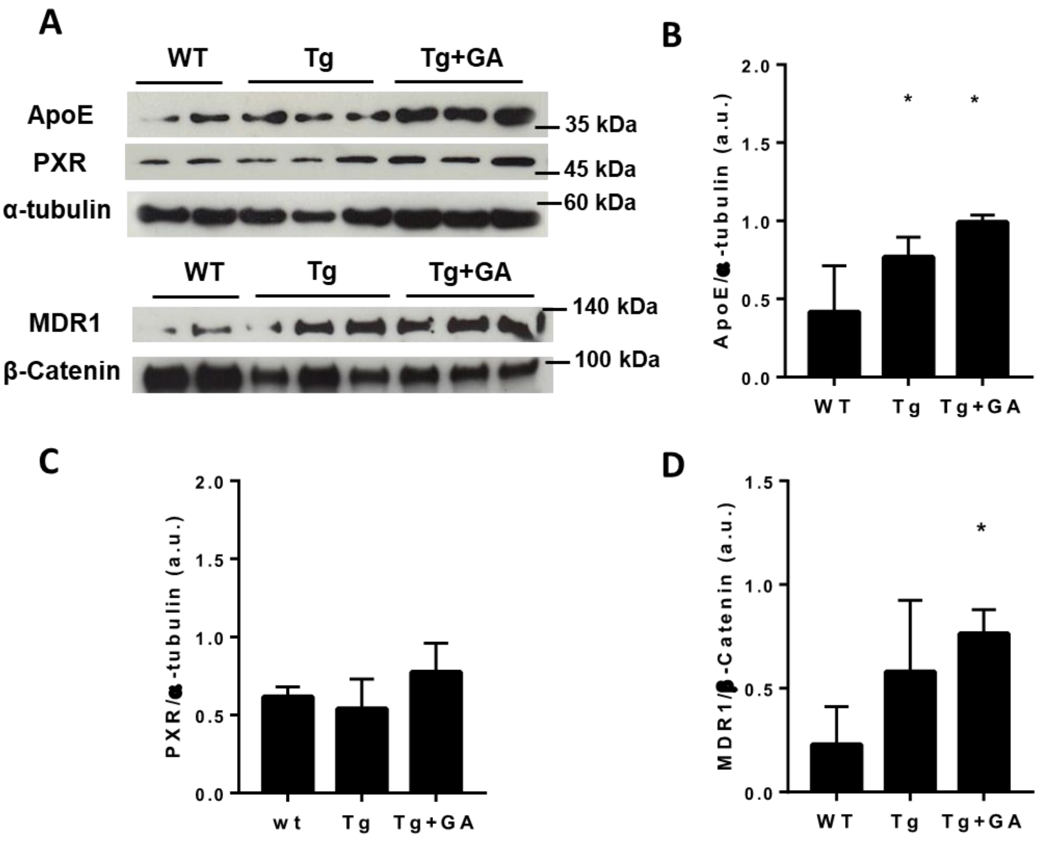

Suppl. Fig. S3

Suppl. Figure S3. Immunoblot of target genes in the hippocampus of TgCRND8 mice treated with GA. ApoE (B), PXR (C) and MDR1 (D) were investigated in the hippocampus of TgCRND8 mice after oral administration of GA (200 mg/kg; Tg+GA group) or vehicle (olive oil; Tg group) for ten (10) days. Corresponding histology of brain sections is shown in Figure 4. Other details on these animal experiments are reported in Experimental procedures. $*p<0.05$ vs wt.

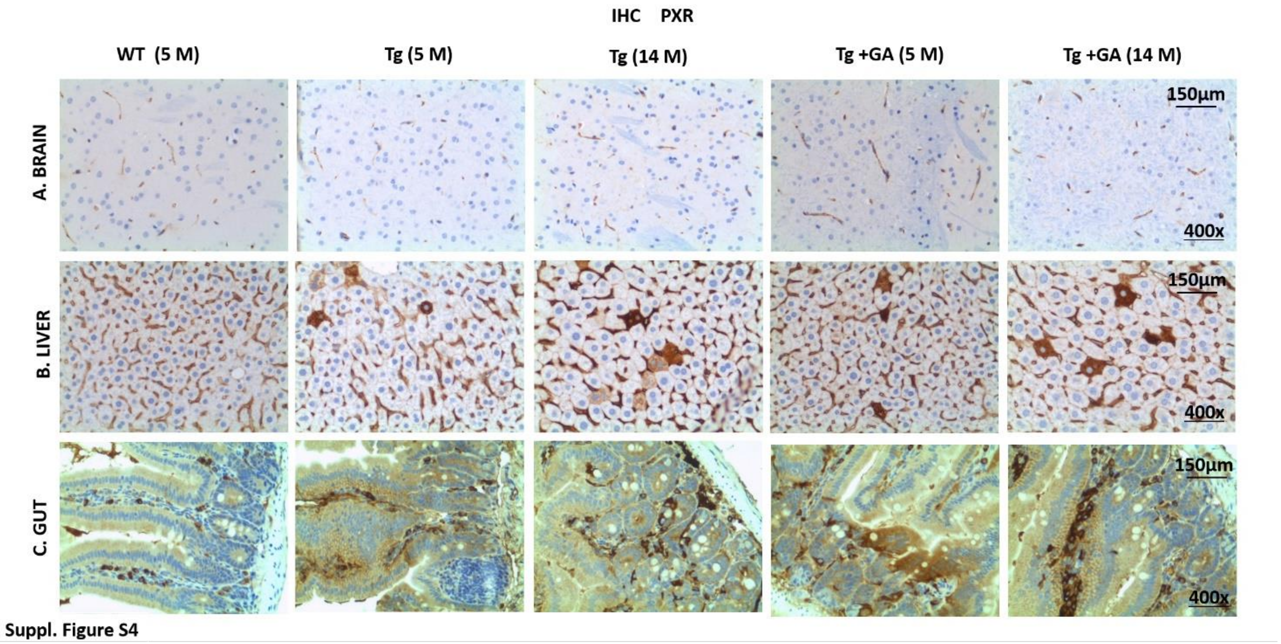

Suppl. Figure S4. Immunohistochemistry (IHC) of PXR in mice brain (A), liver (B) and gut (C). Mice groups and experimental conditions were as in Suppl. Figure S3 and Figure 4. The age of the animal was: 5- month old (5M) or 14-month-old (14M).

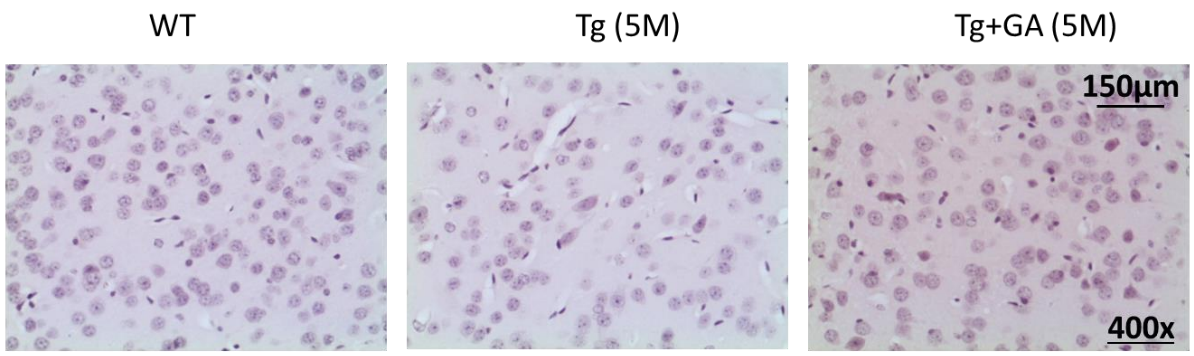

Suppl. Figure S5

Suppl. Figure S5. Control images of Congo Red histology experiments performed in brain sections of TgCRND8 mice treated with GA. Corresponding images are reported in Figure 4. Determinations were performed after oral administration of GA (200 mg/kg; Tg+GA group) or vehicle (olive oil; Tg group) for ten (10) days. Other details on these animal experiments are reported in Experimental procedures and in Figure 4.
